# Supplementary material for: A Prospective Observational Study on Short and Long-Term Outcomes of COVID-19 Patients with Acute Hypoxic Respiratory Failure Treated with High-Flow Nasal Cannula
Source: J Clin Med. 2023 Feb 4;12(4):1249. doi: 10.3390/jcm12041249 (PMC9965220; doi:10.3390/jcm12041249)
Supplement: Supplementary file 1 [file jcm-12-01249-s001.zip › jcm-2114481-supplementary.pdf]

SUPPLEMENTARY MATERIAL

Phone 6-month follow-up questionnaire and Katz index of independence in activity of daily living

1. Is the patient still alive? **yes/no**
2. Did you have a job before the hospitalization? **yes/no**
3. Are you back to work? **yes/no**
4. How many days after the discharge from the hospital did you start to work?
5. After you returned home, do you suffer of problems you did not have before? **yes/no**
6. Is your quality of life the same as the one before the hospitalization? **yes/no**
7. Are you able to walk like you used before or perform activities (walk 1-2 flat of stairs) like you used to? **yes/no**
8. Do you take any new medications you did not take before COVID-19 infection? **yes/no**
9. Were you diagnosed with new medical problems you did not have before COVID-19? **yes/no**
10. Were you experiencing some limitations regarding the Independence in Activities of Daily Living before the hospitalization for COVID-19? **yes/no**
11. If not, are experiencing some limitations regarding the Independence in Activities of Daily Living at the date of follow up? **yes/no**
12. If yes, please fill the Katz Index of Independence in Activities of Daily Living below
13. Do you use oxygen supplementation anytime during the day that you did not have before? **yes/no**
14. Do you have breathing problems you did not have before **yes/no**
15. Are you experiencing sensory loss? **yes/no**
16. Do you have any motor function deficit? **yes/no**
17. Have you been admitted to the hospital within twelve months after the hospitalization for COVID-19? **yes/no**
18. If yes, was the reason for your re-hospitalization surgical or medical?
19. Have you been re-admitted to the hospital for reasons related to COVID-19? **yes/no**
20. Have you been intubated during the second admission for COVID-19 related causes? **yes/no**

Table S1. 6-month follow up questionnaire and Katz Index of Independence in Activities of Daily Living

Katz Index of Independence in Activities of Daily Living		
Activities Points (1 or 0)	Independence (1 Point) NO supervision, direction or personal assistance.	Dependence (0 Points) WITH supervision, direction, personal assistance or total care.
BATHING Points: ___________	(1 POINT) Bathes self completely or needs help in bathing only a single part of the body such as the back, genital area or disabled extremity.	(0 POINTS) Need help with bathing more than one part of the body, getting in or out of the tub or shower. Requires total bathing
DRESSING Points: ___________	(1 POINT) Get clothes from closets and drawers and puts on clothes and outer garments complete with fasteners. May have help tying shoes.	(0 POINTS) Needs help with dressing self or needs to be completely dressed.
TOILETING Points: ___________	(1 POINT) Goes to toilet, gets on and off, arranges clothes, cleans genital area without help.	(0 POINTS) Needs help transferring to the toilet, cleaning self or uses bedpan or commode.
TRANSFERRING Points: ___________	(1 POINT) Moves in and out of bed or chair unassisted. Mechanical transfer aids are acceptable	(0 POINTS) Needs help in moving from bed to chair or requires a complete transfer.
CONTINENCE Points: ___________	(1 POINT) Exercises complete self-control over urination and defecation.	(0 POINTS) Is partially or totally incontinent of bowel or bladder
FEEDING Points: ___________	(1 POINT) Gets food from plate into mouth without help. Preparation of food may be done by another person.	(0 POINTS) Needs partial or total help with feeding or requires parenteral feeding.
TOTAL POINTS: _________ SCORING: 6 = High (patient independent) 0 = Low (patient very dependent)		

Table S2. Protocol in use at the Massachusetts General Hospital to guide the decision to start High Flow nasal Cannula treatment. COPD: Chronic Obstructive Pulmonary Disease.

**Patient Selection**
Indications
1. Progressive hypoxemic respiratory failure; P_{aO2}/F_{iO2} < 200 mmHg<br>2. S_{pO2} < 90% with a partial/non-rebreathing mask at ≥ 15L/min<br>3. Patient unable to tolerate oxygen mask (partial rebreathing, non-rebreathing, or high-flow mask), removal of the mask results in significant desaturation or supplemental oxygen requirements prevent oral intake
Contraindications
1. Hypercapnic respiratory failure<br>2. Obstructive sleep apnea<br>3. Tracheostomy<br>4. Acute cardiogenic pulmonary edema<br>5. Stable COPD with baseline increased P_{aCO2}

Table S3. Main demographic variables, comorbidities laboratory results and x-ray findings of the intubated patients. Data are presented as mean [±SD], n [%] or median [interquartile range] unless otherwise specified

	**Alive**	**Deceased**	**P**
**Demographic information, body max index and HFNC duration**			
Subject (intubated), *n* [%]	78 [63.4]	45 [36.6]	
Age, [y]	63 [±13]	72 [11]	<0.01
Gender [female] n [%]	31 [40]	16 [36]	0.65
Race, non-White, n [%]	39 [50]	22 [49]	0.51
Ethnicity non-Hispanic, n [%]	44 [56]	28 [62]	0.75
Body mass index, kg/m^2	30.12 [26.54-34.36]	29.2 [25.45-34.2]	0.62
HFNC duration, hours	13 [3-34]	12 [4-26]	0.93
Time between HFNC start and intubation, hours	14 [4-37]	17 [5-33]	0.37
**Comorbidities**			
No comorbid disease, n [%]	6 [8]	0	0.06
Hypertension, n [%]	57 [75]	37 [88]	0.09
Diabetes mellitus, n [%]	37 [49]	25 [60]	0.29
Chronic kidney disease, n [%]	14 [19]	16 [38]	0.02
Asthma, n [%]	17 [23]	6 [15]	0.27
COPD, n [%]	8 [11]	6 [14]	0.28
Active cancer, n [%]	10 [14]	10 [24]	0.17
HFrEF, n [%]	5 [7]	5 [12]	0.33
Coronary artery disease, n [%]	11 [15]	15 [37]	<0.01
**Vital signs and x-ray findings**			
Oxygen saturation, %	93 [91-96]	93 [89-96]	0.63
Respiratory rate, breaths/min	31.5 [25-37]	30 [25-34]	0.75
FiO_2 before HFNC [%]	90 [78-90]	90 [70-90]	0.6
Heart rate, beats/min	90 [±19.6]	86 [±20.5]	0.32
Mean arterial pressure, mmHg	91 [81-97]	88 [82-99]	0.99
SpO_2/FiO_2	106 [101-134.5]	106.5 [99-126.5]	0.88
Pulmonary x-ray severity [PXS] score, severe [%]*	20 [28]	14 [34]	0.08
**Laboratory values**			
Creatinine	0.89 [0.72-1.30]	1.16 [0.84-1.3]	<0.01
Urea	20.0 [14.0-29.0]	34.0 [20.3-40.0]	<0.01
White blood cells	8.47 [6.08-11.61]	10.64 [6.66-14.23]	0.05
Platelets	239 [179-299]	212 [142-271]	0.21
Bilirubin	0.5 [0.4-0.6]	0.5 [0.3-0.7]	0.90
C-reactive protein	144.1 [78.1-219.1]	123.1 [49.7-219]	0.18
C_{RS} after intubation, ml/cmH2O	36 [29-44]	30 [25-34]	<0.01
PaO_2/FiO_2 after intubation, mmHg	160 [107-240]	152 [107-192]	0.32
CRS 24 hours after intubation, ml/cmH2O	34 [28-43]	28 [24-34]	<0.01
PaO_2/FiO_2 24 hours after intubation, mmHg	215 [178-246]	204 [169-250]	0.82

*Assessed before HFNC.
Abbreviations: COPD: Chronic obstructive pulmonary disease; C_{rs}: Compliance of the respiratory system; HFNC: High flow nasal cannula; HFrEF: Heart failure with reduced ejection fraction.

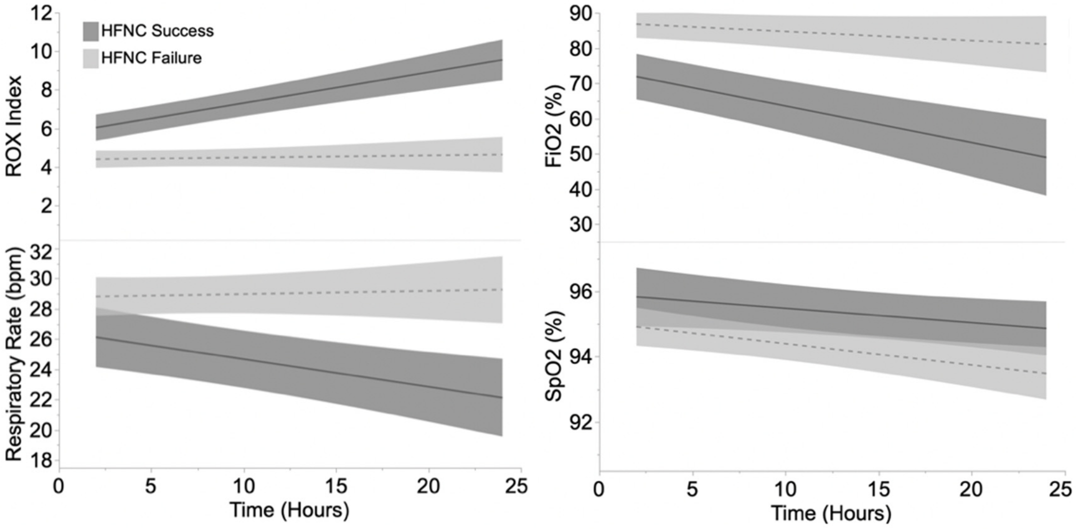

Figure S1. The continuous (non-intubated) and dotted (intubated, **early**-failure) lines show the values predicted by a linear mixed model. We defined early HFNC failure if the intubation occurred within 48 hours since the initiation of HFNC. ROX Index (panel A), Respiratory Rate (RR, panel B), inhaled fraction of oxygen (FiO2, panel C), and peripheral saturation of oxygen (SpO2, panel D) during High Flow Nasal Canula (HFNC) support

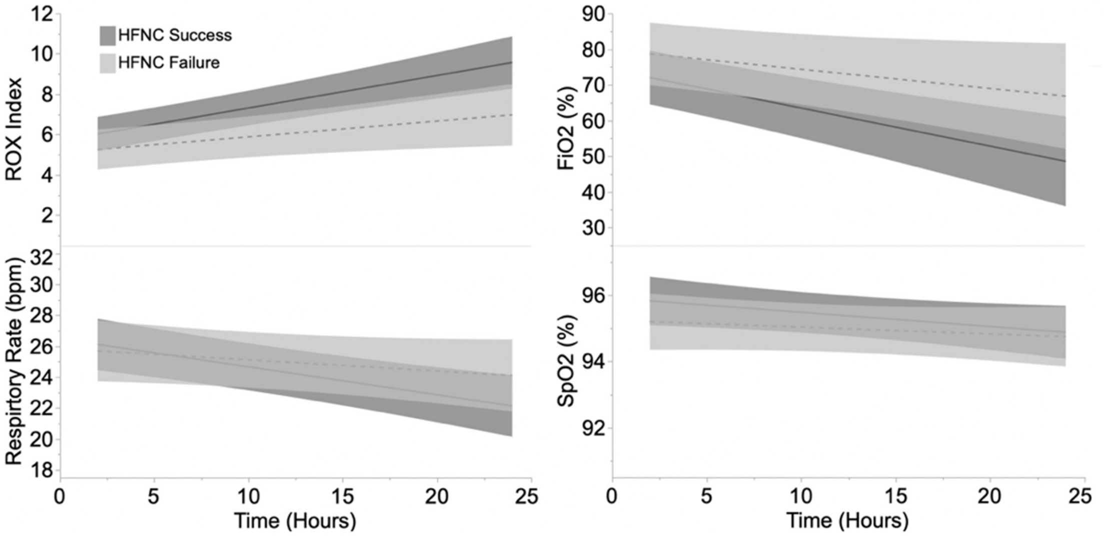

Figure S2. The continuous (HFNC success) and dotted (HFNC failure, **late**-failure) lines show the values predicted by a linear mixed model. We defined late HFNC failure if the intubation occurred after more than 48 hours since the initiation of HFNC. ROX Index (panel A), Respiratory Rate (RR, panel B), inhaled fraction of oxygen (FiO2, panel C), and peripheral saturation of oxygen (SpO2, panel D) during High Flow Nasal Canula (HFNC) support.

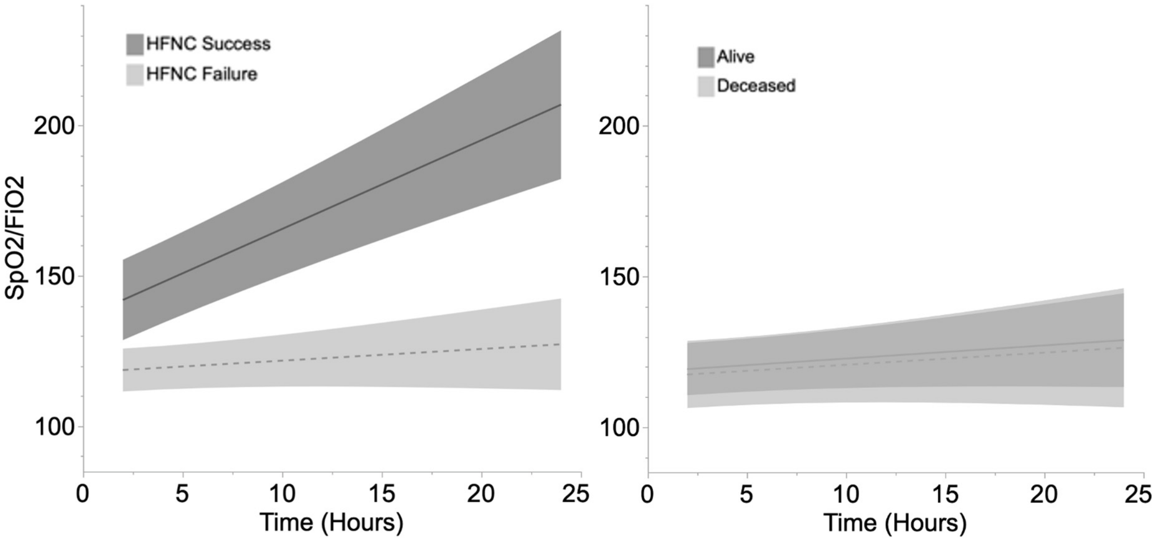

Figure S3. Values of SpO_2/FiO_2 (%) during High Flow Nasal Canula (HFNC) in non-intubated and intubated patients (A) and SpO_2/FiO_2(%) during HFNC in intubated patients: survivor vs non-survivors. Trajectory predicted with a linear mixed model with fixed effects on time and different groups (intubation in panel A, outcomes in panel B) and random effect on the patients.
